# 2-{2-[(*E*)-(2-Benzoyl­hydrazin-1-yl­idene)meth­yl]phen­oxy}acetic acid

**DOI:** 10.1107/S1600536812028735

**Published:** 2012-06-30

**Authors:** Hoong-Kun Fun, Tze Shyang Chia, Ahmed M. Alafeefy, Hatem A. Abdel-Aziz

**Affiliations:** a X-ray Crystallography Unit, School of Physics, Universiti Sains Malaysia, 11800 USM, Penang, Malaysia; b Department of Pharmaceutical Chemistry, College of Pharmacy, Salman Bin Abdulaziz University, PO Box 173, Alkharj 11942, Saudi Arabia; c Department of Pharmaceutical Chemistry, College of Pharmacy, King Saud University, PO Box 2457, Riyadh 11451, Saudi Arabia

## Abstract

In the title compound, C_16_H_14_N_2_O_4_, the dihedral angle between the aromatic rings is 12.45 (6)°. The central C(=O)—N—N=C bridge is roughly planar (r.m.s. deviation = 0.0346 Å) and makes dihedral angles of 13.01 (7) and 0.56 (7)° with the attached phenyl and benzene rings, respectively. The acetic acid unit (r.m.s. deviation = 0.0066 Å) is twisted from its attached benzene ring [dihedral angle = 19.48 (6)°]. In the crystal, mol­ecules are linked by O—H⋯(O,N), N—H⋯O and C—H⋯O hydrogen bonds into sheets lying parallel to the *bc* plane. A weak aromatic π–π stacking inter­action is also observed [centroid–centroid distance = 3.7330 (7) Å].

## Related literature

For background to the biological activity of hydrazones, see: Abdel-Aziz & Mekawey (2009 ▶). For a related structure, see: Rassem *et al.* (2012 ▶). For further synthetic details, see: Desai *et al.* (2000 ▶). For the stability of the temperature controller used in the data collection, see: Cosier & Glazer (1986 ▶).
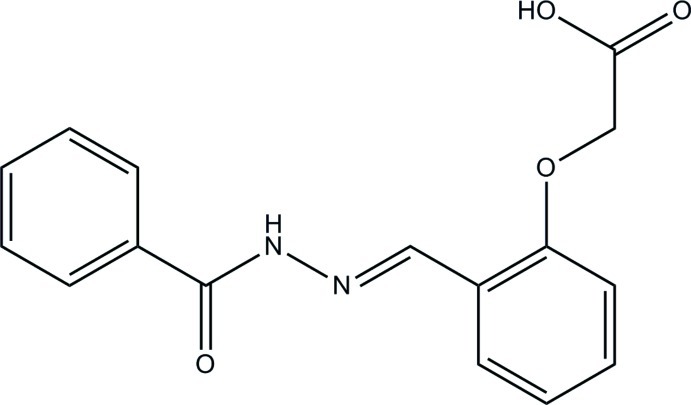


## Experimental

### 

#### Crystal data

C_16_H_14_N_2_O_4_
*M* *_r_* = 298.29
Monoclinic, 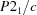

*a* = 12.2173 (7) Å
*b* = 7.8523 (5) Å
*c* = 15.6025 (9) Å
β = 108.577 (1)°
*V* = 1418.82 (15) Å^3^
*Z* = 4
Mo *K*α radiation
μ = 0.10 mm^−1^
*T* = 100 K
0.32 × 0.21 × 0.14 mm

#### Data collection

Bruker APEX DUO CCD diffractometer
Absorption correction: multi-scan (*SADABS*; Bruker, 2009 ▶) *T*_min_ = 0.969, *T*_max_ = 0.986
14301 measured reflections
4122 independent reflections
3463 reflections with *I* > 2σ(*I*)
*R*_int_ = 0.024

#### Refinement

*R*[*F* ^2^ > 2σ(*F* ^2^)] = 0.040
*wR*(*F* ^2^) = 0.124
*S* = 1.07
4122 reflections
203 parameters
H atoms treated by a mixture of independent and constrained refinement
Δρ_max_ = 0.42 e Å^−3^
Δρ_min_ = −0.25 e Å^−3^

### 

Data collection: *APEX2* (Bruker, 2009 ▶); cell refinement: *SAINT* (Bruker, 2009 ▶); data reduction: *SAINT*; program(s) used to solve structure: *SHELXTL* (Sheldrick, 2008 ▶); program(s) used to refine structure: *SHELXTL*; molecular graphics: *SHELXTL*; software used to prepare material for publication: *SHELXTL* and *PLATON* (Spek, 2009 ▶).

## Supplementary Material

Crystal structure: contains datablock(s) global, I. DOI: 10.1107/S1600536812028735/hb6868sup1.cif

Structure factors: contains datablock(s) I. DOI: 10.1107/S1600536812028735/hb6868Isup2.hkl

Supplementary material file. DOI: 10.1107/S1600536812028735/hb6868Isup3.cml

Additional supplementary materials:  crystallographic information; 3D view; checkCIF report

## Figures and Tables

**Table 1:** Hydrogen-bond geometry (Å, °)

*D*—H⋯*A*	*D*—H	H⋯*A*	*D*⋯*A*	*D*—H⋯*A*
O3—H1*O*3⋯O1^i^	0.88	1.99	2.7194 (14)	139
O3—H1*O*3⋯N2^i^	0.88	2.30	3.0464 (12)	142
N1—H1*N*1⋯O4^ii^	0.925 (17)	2.009 (16)	2.9131 (13)	165.4 (16)
C5—H5*A*⋯O4^ii^	0.93	2.55	3.4058 (16)	153
C11—H11*A*⋯O1^iii^	0.93	2.51	3.3791 (13)	155
C15—H15*A*⋯O1^iv^	0.97	2.59	3.5434 (15)	167

Symmetry codes: (i) 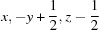; (ii) 
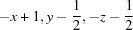
; (iii) 
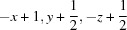
; (iv) 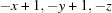.

**Fig. 1.**
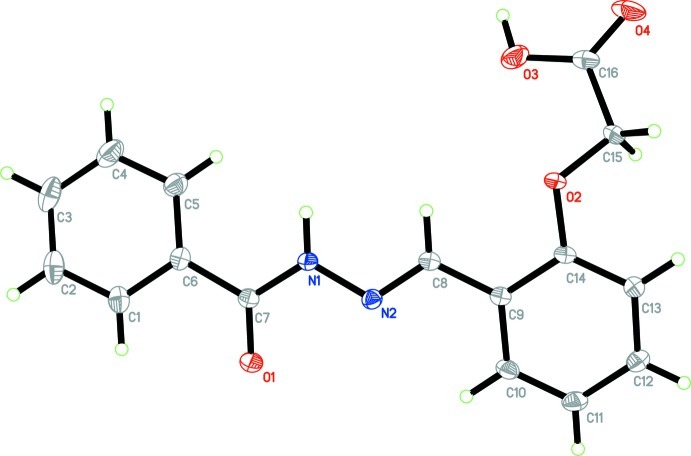
The molecular structure of the title compound with 50% probability displacement ellipsoids.

**Fig. 2.**
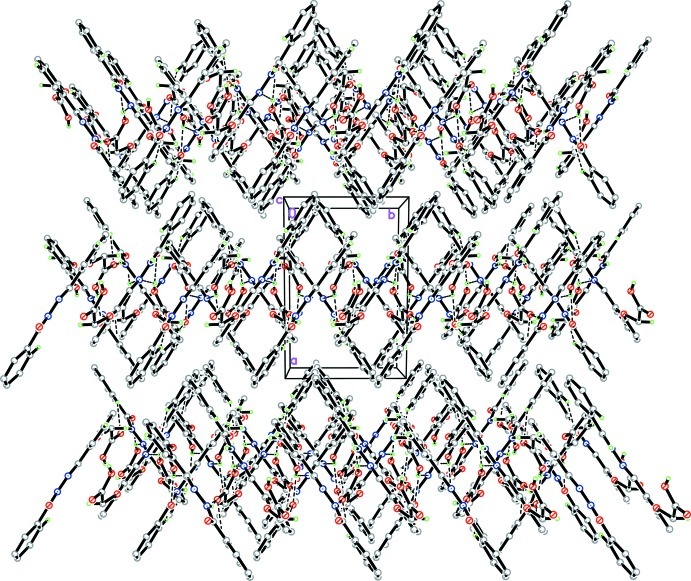
The crystal packing of the title compound. The dashed lines represent the hydrogen bonds. For clarity sake, hydrogen atoms not involved in hydrogen bonding have been omitted.

## supplementary crystallographic information

### Comment

In continuation to our interest in the chemistry and biological activities of hydrazones (Abdel-Aziz *et al.*, 2009), we now report herein the crystal structure of the title compound.

The asymmetric unit of the title compound is shown in Fig. 1. The C1–C6 and C9–C14 benzene rings are slightly twisted from each other as indicated by the dihedral angle of 12.45 (6)°. The central C7(═O1)—N1—N2═C8 bridge is nearly planar [r.m.s. deviation = 0.0346 Å], extended in a zigzag conformation and makes dihedral angles of 13.01 (7) and 0.56 (7)° with the C1–C6 and C9–C14 benzene rings, respectively. The acetic acid unit [C15/C16/O3/O4; r.m.s. deviation = 0.0066 Å] is twisted from its attached C9–C14 benzene ring with dihedral angle of 19.48 (6)°. Bond lengths and angles are comparable to those in a related structure (Rassem *et al.*, 2012).

In the crystal (Fig. 2), molecules are linked by O3—H1O3···O1, O3—H1O3···N2, N1—H1N1···O4, C5—H5A···O4, C11—H11A···O1 and C15—H15A···O1 hydrogen bonds (Table 1) into two-dimensional networks parallel to *bc* plane. π-π interaction is also observed with *Cg*1···*Cg*2 distance of 3.7330 (7) Å [symmentry operator = 1 - *x*,-*y*,-*z*], where *Cg*1 and *Cg*2 are the centroids of C1–C6 and C9–C14 rings, respectively.

### Experimental

The title compound was prepared by heating 2-(2-formylphenoxy)acetic acid with benzohydrazide in absolute ethanol for 4 h (Desai *et al.*, 2000). Colourless blocks were obtained by slow evaporation from EtOH/DMF.

### Refinement

The atoms H1O3 and H1N1 were located in a difference fourier map. Atom H1O3 was then fixed at its found location [O3—H1O3 = 0.8843 Å] and refined using a riding model with *U*_iso_(H) = 1.2*U*_eq_(O), whereas the atom H1N1 was refined freely [N1—H1N1 = 0.924 (17) Å]. The remaining H atoms were positioned geometrically [C—H = 0.93 and 0.97 Å] and refined with *U*_iso_(H) = 1.2*U*_eq_(C).

### Crystal data

**Table:**

C_16_H_14_N_2_O_4_	*F*(000) = 624
*M**_r_* = 298.29	*D*_x_ = 1.396 Mg m^−^^3^
Monoclinic, *P*2_1_/*c*	Mo *K*α radiation, λ = 0.71073 Å
Hall symbol: -P 2ybc	Cell parameters from 5671 reflections
*a* = 12.2173 (7) Å	θ = 2.8–30.1°
*b* = 7.8523 (5) Å	µ = 0.10 mm^−^^1^
*c* = 15.6025 (9) Å	*T* = 100 K
β = 108.577 (1)°	Block, colourless
*V* = 1418.82 (15) Å^3^	0.32 × 0.21 × 0.14 mm
*Z* = 4	

### Data collection

**Table:**

Bruker APEX DUO CCD diffractometer	4122 independent reflections
Radiation source: fine-focus sealed tube	3463 reflections with *I* > 2σ(*I*)
Graphite monochromator	*R*_int_ = 0.024
φ and ω scans	θ_max_ = 30.1°, θ_min_ = 2.8°
Absorption correction: multi-scan (*SADABS*; Bruker, 2009)	*h* = −17→16
*T*_min_ = 0.969, *T*_max_ = 0.986	*k* = −11→10
14301 measured reflections	*l* = −22→22

### Refinement

**Table:**

Refinement on *F*^2^	Primary atom site location: structure-invariant direct methods
Least-squares matrix: full	Secondary atom site location: difference Fourier map
*R*[*F*^2^ > 2σ(*F*^2^)] = 0.040	Hydrogen site location: inferred from neighbouring sites
*wR*(*F*^2^) = 0.124	H atoms treated by a mixture of independent and constrained refinement
*S* = 1.07	*w* = 1/[σ^2^(*F*_o_^2^) + (0.0673*P*)^2^ + 0.394*P*] where *P* = (*F*_o_^2^ + 2*F*_c_^2^)/3
4122 reflections	(Δ/σ)_max_ < 0.001
203 parameters	Δρ_max_ = 0.42 e Å^−^^3^
0 restraints	Δρ_min_ = −0.25 e Å^−^^3^

### Special details

**Table:**

Experimental. The crystal was placed in the cold stream of an Oxford Cryosystems Cobra open-flow nitrogen cryostat (Cosier & Glazer, 1986) operating at 100.0 (1) K.
Geometry. All e.s.d.'s (except the e.s.d. in the dihedral angle between two l.s. planes) are estimated using the full covariance matrix. The cell e.s.d.'s are taken into account individually in the estimation of e.s.d.'s in distances, angles and torsion angles; correlations between e.s.d.'s in cell parameters are only used when they are defined by crystal symmetry. An approximate (isotropic) treatment of cell e.s.d.'s is used for estimating e.s.d.'s involving l.s. planes.
Refinement. Refinement of *F*^2^ against ALL reflections. The weighted *R*-factor *wR* and goodness of fit *S* are based on *F*^2^, conventional *R*-factors *R* are based on *F*, with *F* set to zero for negative *F*^2^. The threshold expression of *F*^2^ > σ(*F*^2^) is used only for calculating *R*-factors(gt) *etc*. and is not relevant to the choice of reflections for refinement. *R*-factors based on *F*^2^ are statistically about twice as large as those based on *F*, and *R*- factors based on ALL data will be even larger.

### Fractional atomic coordinates and isotropic or equivalent isotropic displacement parameters (Å^2^)

**Table:**

	*x*	*y*	*z*	*U*_iso_*/*U*_eq_	
O1	0.70132 (7)	0.06520 (12)	0.21655 (5)	0.0260 (2)	
O2	0.35596 (7)	0.38508 (10)	−0.16701 (5)	0.01834 (17)	
O3	0.48909 (8)	0.40025 (12)	−0.26386 (6)	0.0273 (2)	
H1O3	0.5341	0.4088	−0.2982	0.033*	
O4	0.35806 (9)	0.53387 (12)	−0.37939 (6)	0.0305 (2)	
N1	0.64695 (7)	0.09189 (11)	0.06501 (6)	0.01427 (18)	
N2	0.55352 (7)	0.18816 (11)	0.06821 (6)	0.01471 (18)	
C1	0.88465 (10)	−0.14596 (14)	0.22331 (7)	0.0202 (2)	
H1A	0.8587	−0.1404	0.2732	0.024*	
C2	0.98417 (10)	−0.23668 (15)	0.22871 (9)	0.0262 (2)	
H2A	1.0239	−0.2935	0.2818	0.031*	
C3	1.02450 (10)	−0.24288 (17)	0.15531 (10)	0.0304 (3)	
H3A	1.0920	−0.3018	0.1594	0.036*	
C4	0.96375 (11)	−0.16065 (17)	0.07552 (10)	0.0299 (3)	
H4A	0.9907	−0.1654	0.0261	0.036*	
C5	0.86271 (10)	−0.07092 (15)	0.06869 (8)	0.0221 (2)	
H5A	0.8220	−0.0170	0.0149	0.027*	
C6	0.82339 (9)	−0.06286 (13)	0.14308 (7)	0.0162 (2)	
C7	0.71940 (9)	0.03547 (13)	0.14467 (7)	0.01526 (19)	
C8	0.49302 (8)	0.25514 (13)	−0.00687 (6)	0.01392 (19)	
H8A	0.5128	0.2371	−0.0590	0.017*	
C9	0.39275 (8)	0.36004 (13)	−0.01025 (6)	0.01350 (19)	
C10	0.36525 (9)	0.40247 (14)	0.06745 (7)	0.0177 (2)	
H10A	0.4114	0.3619	0.1233	0.021*	
C11	0.27065 (10)	0.50378 (15)	0.06311 (7)	0.0220 (2)	
H11A	0.2542	0.5322	0.1156	0.026*	
C12	0.20069 (10)	0.56235 (15)	−0.02047 (7)	0.0206 (2)	
H12A	0.1360	0.6278	−0.0239	0.025*	
C13	0.22630 (9)	0.52432 (14)	−0.09895 (7)	0.0169 (2)	
H13A	0.1793	0.5643	−0.1546	0.020*	
C14	0.32297 (8)	0.42582 (13)	−0.09354 (6)	0.01383 (19)	
C15	0.31286 (9)	0.48856 (14)	−0.24511 (7)	0.0180 (2)	
H15A	0.3096	0.6062	−0.2272	0.022*	
H15B	0.2352	0.4528	−0.2792	0.022*	
C16	0.38970 (10)	0.47431 (14)	−0.30367 (7)	0.0196 (2)	
H1N1	0.6582 (14)	0.066 (2)	0.0106 (11)	0.030 (4)*	

### Atomic displacement parameters (Å^2^)

**Table:**

	*U*^11^	*U*^22^	*U*^33^	*U*^12^	*U*^13^	*U*^23^
O1	0.0283 (4)	0.0379 (5)	0.0125 (3)	0.0126 (4)	0.0072 (3)	0.0028 (3)
O2	0.0218 (4)	0.0241 (4)	0.0112 (3)	0.0062 (3)	0.0082 (3)	0.0036 (3)
O3	0.0275 (4)	0.0309 (5)	0.0311 (4)	0.0042 (4)	0.0200 (4)	0.0053 (4)
O4	0.0533 (6)	0.0261 (4)	0.0172 (4)	0.0037 (4)	0.0184 (4)	0.0046 (3)
N1	0.0150 (4)	0.0165 (4)	0.0117 (4)	0.0012 (3)	0.0048 (3)	−0.0007 (3)
N2	0.0143 (4)	0.0158 (4)	0.0141 (4)	0.0005 (3)	0.0046 (3)	−0.0013 (3)
C1	0.0198 (5)	0.0167 (5)	0.0203 (5)	0.0007 (4)	0.0010 (4)	−0.0014 (4)
C2	0.0198 (5)	0.0185 (5)	0.0328 (6)	0.0023 (4)	−0.0023 (4)	−0.0009 (4)
C3	0.0182 (5)	0.0232 (6)	0.0491 (8)	0.0042 (4)	0.0097 (5)	−0.0008 (5)
C4	0.0269 (6)	0.0282 (6)	0.0414 (7)	0.0040 (5)	0.0202 (5)	−0.0003 (5)
C5	0.0216 (5)	0.0227 (5)	0.0247 (5)	0.0018 (4)	0.0111 (4)	0.0008 (4)
C6	0.0143 (4)	0.0145 (4)	0.0187 (5)	−0.0008 (4)	0.0037 (4)	−0.0016 (4)
C7	0.0160 (4)	0.0160 (4)	0.0138 (4)	−0.0006 (4)	0.0047 (3)	−0.0003 (3)
C8	0.0153 (4)	0.0142 (4)	0.0128 (4)	−0.0021 (3)	0.0051 (3)	−0.0007 (3)
C9	0.0147 (4)	0.0133 (4)	0.0127 (4)	−0.0022 (3)	0.0047 (3)	−0.0007 (3)
C10	0.0218 (5)	0.0194 (5)	0.0122 (4)	0.0003 (4)	0.0055 (4)	0.0000 (4)
C11	0.0273 (5)	0.0247 (5)	0.0170 (5)	0.0037 (4)	0.0114 (4)	−0.0021 (4)
C12	0.0206 (5)	0.0225 (5)	0.0214 (5)	0.0044 (4)	0.0103 (4)	−0.0003 (4)
C13	0.0164 (4)	0.0188 (5)	0.0157 (4)	0.0015 (4)	0.0051 (4)	0.0018 (4)
C14	0.0159 (4)	0.0152 (4)	0.0115 (4)	−0.0017 (3)	0.0059 (3)	−0.0007 (3)
C15	0.0187 (5)	0.0234 (5)	0.0120 (4)	0.0017 (4)	0.0050 (4)	0.0040 (4)
C16	0.0289 (6)	0.0170 (5)	0.0160 (4)	−0.0020 (4)	0.0117 (4)	−0.0007 (4)

### Geometric parameters (Å, º)

**Table:**

O1—C7	1.2325 (12)	C5—C6	1.3918 (15)
O2—C14	1.3686 (11)	C5—H5A	0.9300
O2—C15	1.4191 (12)	C6—C7	1.4936 (14)
O3—C16	1.3107 (15)	C8—C9	1.4631 (14)
O3—H1O3	0.8843	C8—H8A	0.9300
O4—C16	1.2136 (13)	C9—C10	1.3975 (13)
N1—C7	1.3504 (13)	C9—C14	1.4059 (13)
N1—N2	1.3831 (11)	C10—C11	1.3869 (15)
N1—H1N1	0.924 (17)	C10—H10A	0.9300
N2—C8	1.2821 (13)	C11—C12	1.3901 (16)
C1—C2	1.3884 (16)	C11—H11A	0.9300
C1—C6	1.3985 (15)	C12—C13	1.3895 (14)
C1—H1A	0.9300	C12—H12A	0.9300
C2—C3	1.3843 (19)	C13—C14	1.3916 (14)
C2—H2A	0.9300	C13—H13A	0.9300
C3—C4	1.389 (2)	C15—C16	1.5084 (14)
C3—H3A	0.9300	C15—H15A	0.9700
C4—C5	1.3956 (16)	C15—H15B	0.9700
C4—H4A	0.9300		
			
C14—O2—C15	117.23 (8)	C9—C8—H8A	120.1
C16—O3—H1O3	109.9	C10—C9—C14	118.14 (9)
C7—N1—N2	116.90 (8)	C10—C9—C8	122.20 (9)
C7—N1—H1N1	121.8 (10)	C14—C9—C8	119.62 (8)
N2—N1—H1N1	121.3 (10)	C11—C10—C9	121.42 (9)
C8—N2—N1	115.68 (8)	C11—C10—H10A	119.3
C2—C1—C6	120.16 (11)	C9—C10—H10A	119.3
C2—C1—H1A	119.9	C10—C11—C12	119.29 (9)
C6—C1—H1A	119.9	C10—C11—H11A	120.4
C3—C2—C1	120.23 (11)	C12—C11—H11A	120.4
C3—C2—H2A	119.9	C13—C12—C11	120.79 (10)
C1—C2—H2A	119.9	C13—C12—H12A	119.6
C2—C3—C4	119.72 (11)	C11—C12—H12A	119.6
C2—C3—H3A	120.1	C12—C13—C14	119.41 (9)
C4—C3—H3A	120.1	C12—C13—H13A	120.3
C3—C4—C5	120.67 (12)	C14—C13—H13A	120.3
C3—C4—H4A	119.7	O2—C14—C13	123.35 (9)
C5—C4—H4A	119.7	O2—C14—C9	115.76 (9)
C6—C5—C4	119.43 (11)	C13—C14—C9	120.88 (9)
C6—C5—H5A	120.3	O2—C15—C16	110.21 (9)
C4—C5—H5A	120.3	O2—C15—H15A	109.6
C5—C6—C1	119.78 (10)	C16—C15—H15A	109.6
C5—C6—C7	123.97 (9)	O2—C15—H15B	109.6
C1—C6—C7	116.22 (9)	C16—C15—H15B	109.6
O1—C7—N1	121.36 (9)	H15A—C15—H15B	108.1
O1—C7—C6	120.86 (9)	O4—C16—O3	126.30 (10)
N1—C7—C6	117.77 (9)	O4—C16—C15	119.63 (11)
N2—C8—C9	119.75 (8)	O3—C16—C15	114.03 (9)
N2—C8—H8A	120.1		
			
C7—N1—N2—C8	172.93 (9)	N2—C8—C9—C14	−176.62 (9)
C6—C1—C2—C3	1.17 (17)	C14—C9—C10—C11	1.25 (16)
C1—C2—C3—C4	−1.25 (19)	C8—C9—C10—C11	179.21 (10)
C2—C3—C4—C5	0.4 (2)	C9—C10—C11—C12	0.95 (17)
C3—C4—C5—C6	0.59 (19)	C10—C11—C12—C13	−1.72 (18)
C4—C5—C6—C1	−0.67 (17)	C11—C12—C13—C14	0.25 (17)
C4—C5—C6—C7	177.28 (11)	C15—O2—C14—C13	20.08 (14)
C2—C1—C6—C5	−0.20 (16)	C15—O2—C14—C9	−160.56 (9)
C2—C1—C6—C7	−178.31 (10)	C12—C13—C14—O2	−178.65 (10)
N2—N1—C7—O1	1.68 (15)	C12—C13—C14—C9	2.02 (16)
N2—N1—C7—C6	−177.63 (8)	C10—C9—C14—O2	177.88 (9)
C5—C6—C7—O1	−165.87 (11)	C8—C9—C14—O2	−0.13 (14)
C1—C6—C7—O1	12.14 (15)	C10—C9—C14—C13	−2.74 (15)
C5—C6—C7—N1	13.44 (16)	C8—C9—C14—C13	179.25 (9)
C1—C6—C7—N1	−168.54 (9)	C14—O2—C15—C16	157.84 (9)
N1—N2—C8—C9	−179.66 (8)	O2—C15—C16—O4	169.40 (10)
N2—C8—C9—C10	5.46 (15)	O2—C15—C16—O3	−12.74 (13)

### Hydrogen-bond geometry (Å, º)

**Table:**

*D*—H···*A*	*D*—H	H···*A*	*D*···*A*	*D*—H···*A*
O3—H1*O*3···O1^i^	0.88	1.99	2.7194 (14)	139
O3—H1*O*3···N2^i^	0.88	2.30	3.0464 (12)	142
N1—H1*N*1···O4^ii^	0.925 (17)	2.009 (16)	2.9131 (13)	165.4 (16)
C5—H5*A*···O4^ii^	0.93	2.55	3.4058 (16)	153
C11—H11*A*···O1^iii^	0.93	2.51	3.3791 (13)	155
C15—H15*A*···O1^iv^	0.97	2.59	3.5434 (15)	167

Symmetry codes: (i) *x*, −*y*+1/2, *z*−1/2; (ii) −*x*+1, *y*−1/2, −*z*−1/2; (iii) −*x*+1, *y*+1/2, −*z*+1/2; (iv) −*x*+1, −*y*+1, −*z*.
